# Associations between dietary n-6 and n-3 fatty acids and arachidonic acid compositions in plasma and erythrocytes in young and elderly Japanese volunteers

**DOI:** 10.1186/1476-511X-10-138

**Published:** 2011-08-13

**Authors:** Terue Kawabata, Satoko Hirota, Tomoko Hirayama, Naoko Adachi, Yoshinori Kaneko, Noriko Iwama, Keiko Kamachi, Eiji Araki, Hiroshi Kawashima, Yoshinobu Kiso

**Affiliations:** 1 Faculty of Nutrition, Kagawa Nutrition University, 3-9-21 Chiyoda, Sakado, Saitama 350-0288, Japan; 2 Department of Food and Nutrition, Junior College of Kagawa Nutrition University, 3-24-3 Komagome, Toshima-ku, Tokyo 170-8481, Japan; 3 Institute for Health Care Science, Suntory Wellness Ltd., 1-1-1 Wakayamadai, Shimamoto, Osaka 618-8503, Japan

## Abstract

**Background:**

We reported that the compositions of arachidonic acid (ARA) in erythrocytes and plasma phospholipids (PL) in the elderly were lower than those in the young, though the ARA intake was nearly identical.

**Objective:**

We further analyzed data in four study groups with different ages and sexes, and determined that the blood ARA levels were affected by the kinds of dietary fatty acids ingested.

**Methods:**

One hundred and four healthy young and elderly volunteers were recruited. Dietary records together with photographic records from 28 consecutive days were reviewed and the fatty acid composition in plasma lipid fractions and erythrocyte PL was analyzed.

**Results:**

No correlations for ARA between dietary fatty acids and blood lipid fractions were observed. A significant negative correlation between eicosapentaenoic acid (EPA) + docosahexaenoic acid (DHA) intake and ARA composition in erythrocyte PL was observed. ARA composition in erythrocyte PL was significantly lower in elderly subjects than in young subjects, because EPA and DHA intake in elderly subjects was higher than in young subjects. However, after removing the effect of dietary EPA+DHA intake, the ARA composition in erythrocyte PL in elderly subjects was significantly lower than that in young subjects.

**Conclusions:**

Changes in physical conditions with aging influenced the low ARA composition of erythrocyte in elderly subjects in addition to the effects of dietary EPA and DHA.

## Background

Arachidonic acid (ARA), one of the n-6 polyunsaturated fatty acids (PUFA), is the predominant fatty acid (FA) of membrane phospholipids (PL) in mammalian brain and neural tissues [1,2]. ARA rapidly accumulates in the human brain during the growth spurt that starts at the beginning of the third trimester of pregnancy and remains in high demand until about 2 years of age [3,4].

Many studies in the last decade have shown the role of sufficient intake of n-3 PUFA in the prevention of several diseases, particularly coronary heart disease [5-7]. Eicosanoids made from ARA are generally more potent mediators of inflammation, vasoconstriction, and platelet aggregation than those made from the eicosapentaenoic acid (EPA) of n-3 PUFA, so a lower intake of vegetable oil containing abundant linoleic acid (LA), the precursor of ARA, has been recommended. However, a recent epidemiological study has indicated that the levels of ARA in blood do not coincide precisely with the incidence of inflammatory diseases [8,9]. The risk of colorectal cancer was inversely associated with erythrocyte compositions of docosahexaenoic acid (DHA), ARA, and PUFA among Japanese men and women [10]. The current study found a U-shaped relationship between blood cell ARA content and acute coronary syndrome case status, which means that the odds for this disease tended to be lower in the second and third quartiles as compared with the first and highest quartiles [11].

Hereafter, we think that our attention will be focused on blood ARA for the associations with disorders; therefore, we should understand how the blood ARA is changed by factors including the discrepancies in diet, sex, and age. Weseler *et al*. [12] reported that dietary supplementation with 200 mg ARA increased ARA in erythrocyte membrane PL in nursing mothers. Our previous data showed that the intake of a capsule containing only 80 mg ARA, which is roughly equivalent to 60% of the usual daily ARA intake, increased the ARA level in all lipid fractions of the blood [13]. Thus, the blood ARA level is markedly increased by dietary intervention with ARA. However, in the studies targeted at a free-living population, previous reports have not coincided in the correlations with dietary ARA and blood ARA composition: these data have not necessarily shown a positive correlation and several data sets have shown a negative correlation or no correlation at all [14-18].

We conducted a dietary survey using dietary records together with photographic records over 28 consecutive days and determined the FA compositions in plasma triacylglycerol (TG), esterified cholesterol (EC), and PL, and erythrocyte membrane PL in four study groups: young men, young women, elderly men, and elderly women. We reported that the compositions of ARA in plasma and erythrocyte PL in the elderly were lower than those in the young, though the ARA intake was nearly identical [19]. In this study, we further analyzed data in four study groups with different ages and sexes, and determined whether the blood ARA levels were affected by the kinds of dietary FA ingested.

## Subjects and methods

### Subjects

The details of the subjects are reported in our previous study [13]. Briefly, groups of healthy subjects in this study were as follows: young men group (YM-G), young women group (YW-G), elderly men group (EM-G), and elderly women group (EW-G). The YM-G consisted of 20 men who were 20 years of age. The YW-G consisted of 30 women who were 20 years of age. The EM-G consisted of 22 men who were 60-75 years of age. The EW-G consisted of 32 women who were 56-73 years of age. The subjects in the four study groups received a verbal explanation of the study and provided written informed consent prior to participating in the study. The study was performed after obtaining approval from the medical ethics review board of Kagawa Nutrition University.

### Dietary assessment

A dietary record was continuously maintained using a written form together with photographic records for 28 consecutive days. Although every investigation was conducted in early summer, the investigation year was 2004 for the young women, 2005 for the elderly men, 2007 for the elderly women, and 2008 for the young men. The details of the dietary survey method are reported in our previous study [20]. Weights of food consumed were estimated from the daily dietary records and food images. The Fifth Revised and Enlarged Edition of the Tables of Fatty Acid Composition [21] in Japan was used as a reference for FA intake.

### Fatty acid analysis of blood lipid fractions

Fasting blood sampling was conducted on the day after completion of the 28-day dietary survey. After blood sampling, the samples were centrifuged to separate plasma and erythrocytes. The preparation of erythrocyte membranes and the analysis of FA compositions were conducted as described previously [13]. Briefly, erythrocytes were washed with saline, and after Tris-HCl buffer was added, they were centrifuged to obtain the erythrocyte membranes. The total FA of the erythrocyte membranes and plasma were extracted, the FA of plasma TG, EC, and PL and erythrocyte membrane PL were separated by thin-layer chromatography and, after transmethylation, the FA composition was analyzed by gas chromatography. The FA compositions were calculated as percentages of the total FA.

### Statistical analysis

The relationships between dietary FA and FA in plasma TG, EC, and PL and erythrocyte membrane PL were examined by Spearman's rank correlation. Statistical differences among the four groups were determined using ANOVA and the Bonferroni post hoc test. We calculated the least square means and 95% confidence intervals of ARA composition in erythrocyte PL using analysis of covariance (ANCOVA) models, with EPA+DHA intakes as the covariables and ARA composition in erythrocyte PL as the dependent variable, because it was possible that dietary EPA+DHA intake affected the ARA composition in erythrocyte PL.

A significant difference in analysis results was observed at *P *< 0.05. We conducted calculations using the Statistical Package for Social Science software (SPSS for Windows, version 17.0; Chicago, IL, USA).

## Results

Spearman's correlation coefficients between dietary EPA, DHA and ARA intake and the corresponding FA composition of plasma TG, EC, and PL and erythrocyte PL in YM-G, YW-G, EM-G, EW-G, and the total subjects group (ALL-G) are shown Table 1. Significant positive correlations between dietary intake and all blood lipid fractions were observed for EPA and DHA in ALL-G. No correlations for ARA were observed between dietary ARA and all blood lipid fractions of YM-G, YW-G, EM-G, and EW-G.

**Table 1:** Spearman's correlation coefficients between dietary EPA, DHA and ARA intake and the corresponding FA composition of plasma TG, EC, and PL and erythrocyte PL in YM-G, YW-G, EM-G, EW-G, and ALL-G

		Plasma TG		Plasma EC		Plasma PL		Erythrocyte PL	
20:5n-3 (EPA)	YM-G	0.617	**	0.611	**	0.665	**	0.693	**
	YW-G	0.188		0.457	*	0.407	*	0.829	***
	EM-G	0.770	***	0.691	***	0.840	***	0.905	***
	EW-G	0.599	***	0.404	*	0.692	***	0.604	***
	All-G	0.658	***	0.654	***	0.704	***	0.852	***
22:6n-3 (DHA)	YM-G	0.392		0.326		0.571	**	0.284	
	YW-G	0.276		0.408	*	0.608	***	0.500	**
	EM-G	0.703	***	0.550	**	0.476	*	0.112	
	EW-G	0.207		0.186		0.220		0.281	
	All-G	0.508	***	0.631	***	0.381	***	0.446	***
20:4n-6 (ARA)	YM-G	-0.269		-0.313		-0.253		-0.095	
	YW-G	0.166		0.248		0.199		-0.047	
	EM-G	0.193		-0.015		0.016		0.053	
	EW-G	-0.035		0.186		0.087		-0.181	
	All-G	0.012		-0.046		-0.085		-0.238	*

Abbreviations: FA, fatty acids; TG, triacylglycerol; EC, esterified cholesterol; PL, phospholipids; EPA, eicosapentaenoic acid; DHA, docosahexaenoic acid; ARA, arachidonic acid; YM-G, young men group; YW-G, young women group; EM-G, elderly men group; EW-G, elderly women group, All-G, all subjects group.

*P < 0.05;**P < 0.01;***P < 0.001.

Spearman's correlation coefficients between dietary EPA and DHA intake and ARA composition of plasma TG, EC, and PL and erythrocyte PL in YM-G, YW-G, EM-G, EW-G, and ALL-G are shown in Table 2. Dietary EPA intakes were significantly and negatively correlated to ARA composition in plasma TG, EC, and PL of ALL-G and erythrocyte PL of YM-G, YW-G, EM-G, EW-G, and ALL-G. Dietary DHA intakes were significantly and negatively correlated to ARA composition in plasma EC and PL of ALL-G and erythrocyte PL of YM-G, YW-G, EM-G, EW-G, and ALL-G.

**Table 2:** Spearman's correlation coefficients between dietary EPA and DHA intake and ARA composition of plasma TG, EC, and PL and erythrocyte PL in YM-G, YW-G, EM-G, EW-G, and ALL-G

		ARA compositions							
		
		Plasma TG		Plasma EC		Plasma PL		Erythrocyte PL	
Dietary intakes									
20:5n-3 (EPA)	YM-G	0.114		-0.189		-0.186		-0.633	**
	YW-G	0.146		-0.285		-0.344		-0.677	***
	EM-G	0.545	**	-0.046		-0.265		-0.505	*
	EW-G	0.003		-0.084		-0.103		-0.503	**
	All-G	-0.203	*	-0.436	***	-0.610	***	-0.815	***
22:6n-3 (DHA)	YM-G	0.030		-0.223		-0.244		-0.618	**
	YW-G	0.193		-0.191		-0.297		-0.599	***
	EM-G	0.542	**	0.046		-0.129		-0.545	**
	EW-G	-0.060		-0.143		-0.172		-0.534	**
	All-G	-0.187		-0.410	***	-0.595	***	-0.811	***

Abbreviations: TG, triacylglycerol; EC, esterified cholesterol; PL, phospholipids; EPA, eicosapentaenoic acid; DHA, docosahexaenoic acid; ARA, arachidonic acid; YM-G, young men group; YW-G, young women group; EM-G, elderly men group; EW-G, elderly women group; All-G, all subjects group.

*P < 0.05; **P < 0.01; ***P < 0.001.

**Figure 1:**
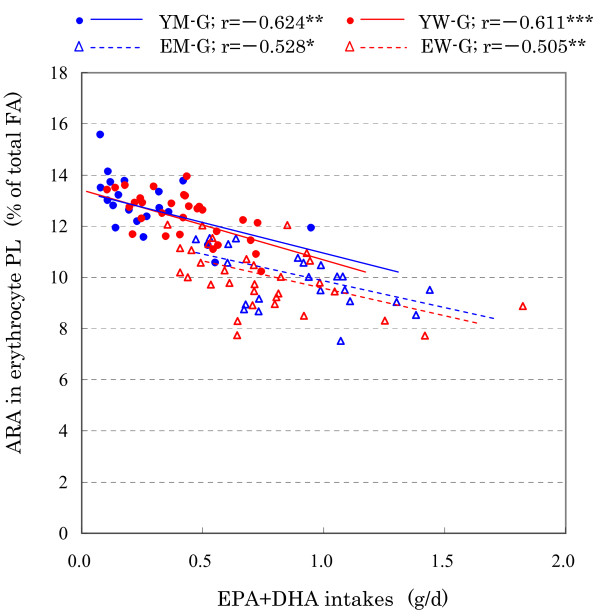
**Scatter plot between dietary EPA+DHA intake and ARA compositions in erythrocyte PL**. Scatter plot between dietary eicosapentaenoic acid (EPA) + docosahexaenoic acid (DHA) intake and arachidonic acid (ARA) compositions in erythrocyte phospholipids (PL) in the young men group (YM-G), young women group (YW-G), elderly men group (EM-G), and elderly women group (EW-G). *p < 0.05; **p < 0.01; ***p < 0.001.

A scatter plot between dietary EPA+DHA intake and ARA compositions in erythrocyte PL is shown in Figure 1. For YM, YW, EM, and EW, EPA+DHA intake and ARA composition in erythrocyte PL, significant negative correlations were observed. Regression lines between men and women overlapped in both the young and elderly groups, while regression lines between young and elderly were parallel in the men and women groups.

Because dietary EPA+DHA intake seems to affect the ARA composition in erythrocyte PL, we analyzed by ANCOVA with EPA+DHA intakes as the covariables. As a result, the F value of common regression was 40.8 (*P *< 0.001), the slope of the regression line was -2.3, and the F value of non-parallelism was 0.54 (*P *= 0.65). As a result, the effect of EPA+DHA intake on ARA compositions in erythrocyte PL was not negligible and the regression lines in YM, YW, EM and EW were parallel. We then calculated the adjusted mean and standard error of the mean (SEM) of ARA composition in erythrocyte PL in YM-G, YW-G, EM-G, and EW-G; these results are shown in Table 3. Adjusted ARA compositions in erythrocyte PL were not significantly different between men and women in both young and elderly subjects; those in the elderly men and women were significantly lower compared with those in the young men and women (*P *< 0.001).

**Table 3:** The mean and SEM of EPA+DHA and ARA intakes and ARA composition in erythrocyte PL among YM-G, YW-G, EM-G, and EW-G

		Dietary intakes (g/d)				Erythrocyte ARA compositions (% of total fatty acids)					
		
		EPA+DHA		ARA		Crude		Adjusted *^2^*		95% confidence limits *^2^*	
YM-G	Mean	0.27		0.15		12.8		12.1		11.6	12.6
	SEM	0.05		0.01		0.3		0.2			
YW-G	Mean	0.41		0.14		12.4		12.0		11.7	12.4
	SEM	0.03		0.01		0.2		0.2			
EM-G	Mean	0.90	*^a, b^*	0.17	*^c^*	9.8	*^a, b^*	10.6	*^a, b^*	10.1	11.0
	SEM	0.06		0.01		0.2		0.2			
EW-G	Mean	0.75	*^a, b^*	0.15		10.0	*^a, b^*	10.3	*^a, b^*	10.0	10.7
	SEM	0.06		0.01		0.2		0.2			

Abbreviations: EPA, eicosapentaenoic acid; DHA, docosahexaenoic acid; ARA, arachidonic acid; PL, phospholipids; YM-G, young men group; YW-G, young women group; EM-G, elderly men group; EW-G, elderly women group.

^*1 *^Significant differences on one-way ANOVA, post ad hoc Bonferroni. *^a^*: different from YM-G, p < 0.001, *^b^*: different from YW-G, p < 0.001 *^c^*: different from YW-G, p < 0.05

*^2 ^*The mean and standard error of the mean (SEM) of adjusted erythrocyte ARA compositions were calculated based on the least squares method by the ANCOVA model. The covariate of this model was estimated from data in which EPA+DHA intake was 0.59 g/d.

## Discussion

We determined the relationships between dietary FA estimated from dietary records together with photographic records over 28 consecutive days and the compositions of ARA in plasma TG, EC, and PL and erythrocyte PL in young men and women and elderly men and women. Our present study showed that: (1) dietary ARA intakes were not correlated with the composition of ARA in erythrocyte PL, but dietary EPA and/or DHA intakes were negatively correlated with the composition of ARA in erythrocyte PL in all subjects groups and (2) after removing the effect of dietary EPA+DHA intake, the ARA composition in erythrocyte PL was significantly lower in elderly subjects than in young subjects.

In previous survey among Japanese people, a negative correlation between ARA intake and serum PL level of ARA was observed [14]. Conversely, Kuriki *et al*. [15] reported that ARA in young and elderly groups demonstrated positive correlations between dietary compositions (wt%) and plasma compositions (wt%). Other studies indicated that the intake of ARA was not significantly related to ARA level in the plasma PL [16,17]. Similarly, Sun *et al*. [18] showed no correlation between the compositions of ARA in plasma and erythrocytes and ARA intake measured with the food-frequency questionnaire in 306 US women. Thus, in the studies targeted at free-living populations, previous reports did not agree on the results of the correlations with dietary ARA and blood ARA composition. In this study, we clearly demonstrated that dietary EPA and/or DHA intakes, but not dietary ARA intakes, markedly affected the ARA compositions in blood ARA levels. This may be one of the reasons that assessment of the ARA level in blood from dietary FA is difficult.

Yanagisawa *et al*. [22] reported an assessment of the serum and erythrocyte FA compositions in groups of Japanese people stratified by age and they indicated that ARA levels of blood lipid fractions in elderly people were lower compared with those in young people. In 530 Yup'ik Eskimos who were 14 to 94 years old, elderly subjects consumed more traditional foods than younger subjects did and in those who consumed traditional foods, the EPA and DHA compositions of red blood cell membranes were significantly higher and ARA composition was significantly lower [23]. Our data also indicated that the compositions of EPA and DHA of plasma and erythrocyte PL in the elderly were significantly higher than those in the young, and those of ARA were significantly lower [19]. We indicated the possibility of the displacement and inhibition of incorporation of ARA by dietary EPA and DHA in blood PL in elderly subjects. In this paper, the negative correlations between EPA+DHA intake and the ARA composition of erythrocyte PL in all subject groups were observed, so the above hypothesis was more strongly supported by our analysis. In addition, we conducted the analysis of ANCOVA, with EPA+DHA intake as the covariable. After removing the effect of dietary EPA+DHA intake, the adjusted ARA composition in erythrocyte PL was significantly lower in elderly subjects than in young subjects. Therefore, in addition to the effects of dietary EPA and DHA, we estimated that changes in physical conditions with aging affected the low ARA composition of erythrocyte in elderly subjects.

Erythrocyte membrane FA composition is affected by diet and is considered to reach a new steady state level 4 to 5 weeks after the establishment of new diet management [24]. We performed a dietary investigation for 28 consecutive days using dietary records together with photographic records to precisely assess habitual FA intakes in order to determine the association between dietary FA and ARA composition in erythrocyte PL. Different conditions, such as a shorter period than our dietary survey period or different dietary survey methods, may not bring about the desired results of the relationship between dietary and erythrocyte ARA. Our present results were obtained under a precise dietary survey for 1 month.

In summary, the ARA levels in blood all lipid fractions, especially in erythrocyte PL, were affected by the amount of EPA and/or DHA intakes. The ARA composition in erythrocyte PL was significantly lower in elderly subjects than in young subjects, because EPA and DHA intakes in elderly subjects were higher than in young subjects. However, after removing the effect of dietary EPA+DHA intake, the adjusted ARA composition of erythrocyte PL in elderly subjects was significantly lower than that in young subjects. Consequently, changes in physical conditions with aging influenced the low ARA composition of erythrocyte in elderly subjects in addition to the effects of dietary EPA and DHA.

## Competing interests

The authors declare that they have no competing interests.

## Authors' contributions

TK conceived the study, participated in the design of the study, acquired data, performed the statistical analysis and drafted the manuscript. SH, TH, NA, YK, NI and KK carried out the survey, measured blood fatty acid compositions, and organized the data. EA, HK and YK participated in the design of the study and helped conducting the study. All authors read and approved the final manuscript.
